# Surveillance of seasonal respiratory viruses among Chilean patients during the COVID‐19 pandemic

**DOI:** 10.1002/hsr2.433

**Published:** 2021-11-23

**Authors:** Luis A. Alonso‐Palomares, C. Joaquín Cáceres, Rodrigo Tapia, Paulina Aguilera‐Cortés, Santiago Valenzuela, Fernando Valiente‐Echeverría, Ricardo Soto‐Rifo, Aldo Gaggero, Gonzalo P. Barriga

**Affiliations:** ^1^ SARS‐CoV‐2 Research Group, Virology Program, Institute of Biomedical Sciences, Faculty of Medicine Universidad de Chile Santiago Chile; ^2^ Laboratory of Molecular and Cellular Virology, Virology Program, Institute of Biomedical Sciences, Faculty of Medicine Universidad de Chile Santiago Chile; ^3^ HIV/AIDS Work Group, Faculty of Medicine Universidad de Chile Santiago Chile; ^4^ Department of Population Health, College of Veterinary Medicine University of Georgia Athens Georgia USA; ^5^ Laboratory of Emerging Viruses, Virology Program, Institute of Biomedical Sciences, Faculty of Medicine Universidad de Chile Santiago Chile; ^6^ Laboratory of Environmental Virology, Virology Program, Institute of Biomedical Sciences, Faculty of Medicine Universidad de Chile Santiago Chile

## ETHICS STATEMENT

The study described here was approved by the Ethics Committee of the Faculty of Medicine at Universidad de Chile (Project N° 036‐2020). The samples were de‐identified and not considered as human samples.

## 1 INTRODUCTION

SARS‐CoV‐2 has generated over 192 million cases worldwide until late July 2021. Non‐pharmaceutical interventions such as confinements and lockdowns started in Chile on March 18, 2020. In Europe, confinements and lockdowns have been accompanied by a decrease in the circulation of other respiratory viruses such as influenza A virus (IAV), influenza B virus (IBV), or respiratory syncytial virus (RSV).^1^ Although changes in circulation patterns of respiratory viruses have been reported, limited information regarding the southern hemisphere is available where the SARS‐CoV‐2 pandemic merged with the winter season.

**FIGURE 1:**
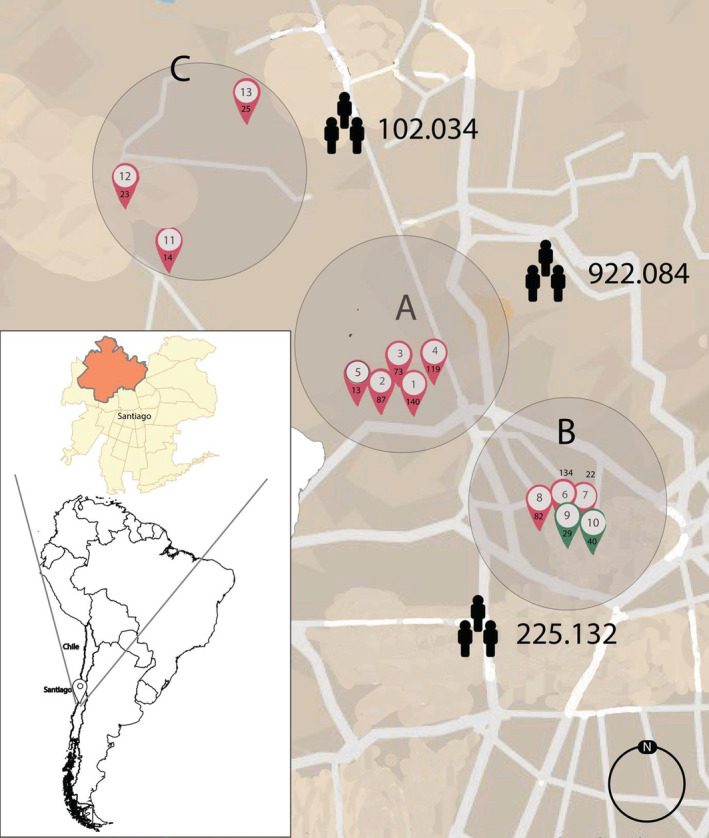
Geographical distribution of the samples analyzed in this study

Few south hemisphere countries reported the same pattern as Europe where non‐pharmaceutical measures began before the winter season,^2^, ^3^ but to the best of our knowledge, no report has been generated containing information from Chile. Here, we collected 11 093 nasopharyngeal‐swab samples (NSS) from 13 healthcare centers belonging part to the northern area of Santiago; the 13 health centers represent all centers to get SARS‐CoV‐2 tested in the selected area in Santiago of Chile, between 1 April and 31 July 2020 (Figure 1). All samples were collected from patients with at least one COVID‐19 symptom. Six thousand seventy‐two samples were determined as positive for SARS‐CoV‐2. Exactly 58.54% was SARS‐CoV‐2 positive showed age range between 18 and 50 years reach a 2.27 incidence rate between 31 and 40 years old (Table S1).

Next, based on geographic location, we divided the positive samples into three groups (A, B, and C). Location (A) contains the highest number of SARS‐CoV‐2 cases, contributing more than 50% of the positive samples analyzed in this study (Figure 1). This high positivity could be explained by the population density of Location A, which contains at least 4‐fold more inhabitants than Locations B and C (Figure 1). Nevertheless, the health centers located at Location B (Figure 1) presented an incidence rate (IR) higher than Location A for SARS‐CoV2 (Table 1); we highlight this result because Location B includes Sub‐locations 9 and 10 (Figure 1), where no positive samples were obtained in the period analyzed. Location C is the farthest location from downtown; however, its IR was 3.82 (Table 1).

**TABLE 1:** Co‐circulation and coinfection of seasonal respiratory viruses together with SARS‐CoV‐2 in the northern area of Santiago, Chile

Location	Healthcare center^a^	SARS‐CoV‐2	IAV	IBV	RSV	HRV	SARS‐CoV‐2/IAV	SARS‐CoV‐2/HRV	Population‐based incidence rates per 1000 population
A	1	82/140 (58.6%)	2/140	0	0	2/140	0	1/140	7.43
A	2	43/89 (49.4%)	0	0	0	1/89	0	0	
A	3	28/76 (38.4%)	0	0	0	0	0	0	
A	4	57/117 (47.9%)	2/117	0	0	0	2/117	0	
A	5	9/13 (69.2%)	0	0	0	0	0	0	
B	6	87/134 (64.9%)	1/134	0	0	0	0	0	20.03
B	7	12/22 (54.56%)	0	0	0	0	0	0	
B	8	48/82 (58.5%)	0	0	0	1/82	0	0	
B	9	0/28 (0%)	1/28	0	0	0	0	0	
B	10	0/40 (0%)	1/40	0	0	0	0	0	
C	11	9/14 (64.3%)	0	0	0	0	0	0	3.82
C	12	7/23 (30.4%)	0	0	0	1/23	0	1/23	
C	13	18/25 (72%)	0	0	0	1/40	0	0	

^a^ This study includes the 800 individuals cohort.

Taken together, our data show a high frequency of positive samples throughout the healthcare centers evaluated, suggesting the population density as a risk factor for SARS‐CoV‐2 transmission because Locations A and B concentrate more population than Location C. These results demonstrate that the 2020 winter season in the northern region of Santiago presented a high incidence of SARS‐CoV‐2 (Tables 1 and 2).

**TABLE 2:** Number distribution of COVID‐19 cases according to age group and sex from 1 April to 31 July 2020 at Santiago of Chile

Age	SARS‐CoV‐2 (%)	SARS‐CoV‐2 number	Incidence rates (IRs)
18‐30	19.65	1232	1.97
31‐40	22.59	1416	2.27
41‐50	16.30	1022	1.64
51‐60	15.39	965	1.54
61‐70	9.16	574	0.92
71‐80	5.90	370	0.59
81‐90	4.07	255	0.41
91‐100	1.77	111	0.18

Gender	Number %	Number	IRs
Male	49.40	3097	4.96
Female	50.44	3162	5.06

*Note*: Six thousand seventy‐two patients are considered positive for SARS‐CoV‐2; however, three patients did not provide any information about age and gender.

Then, we made a random collection of 800 samples (400 positive to SARS‐CoV‐2 and 400 negative) to determine in these samples whether SARS‐CoV‐2 was co‐circulating with other respiratory viruses. We chose predominant respiratory viruses in Santiago (winter season), such as IAV, IBV, RSV, and human rhinovirus (HRV) (Table 1). Adenovirus, parainfluenza, and metapneumovirus were not evaluated because they are considered all‐year viruses.^4^ The results showed three samples with coinfection between IAV and SARS‐CoV‐2 (Table 1). This is congruent to recent studies in Ecuador and Brazil showing a complete decrease of IAV,^5^, ^6^ despite the co‐circulation or coinfection between IAV and SARS‐CoV‐2 observed (Table 1). These results suggest an impact of the non‐pharmaceutical interventions in the circulation of seasonal viruses, as previously reported in Korea and Hong Kong.^7^, ^8^ Next, we evaluated the presence of IAV, IBV, and RSV in the 400 samples reported as negative for SARS‐CoV‐2 where five positive samples were detected for IAV and no positive samples for IBV or RSV were detected, suggesting a circulation of these viruses below 1% considering the number of samples evaluated (Table 1). This is lower than the information from the northern hemisphere, where a range between 2% and 10% has been reported.^9^, ^10^ Taken together, these results suggest a lower co‐circulation of IAV with SARS‐CoV‐2 and co‐circulation below the level of detection of this study for SARS‐CoV‐2 together with IBV or RSV.

Finally, we focused on HRV, responsible for more than 50% of the cold‐like illnesses, with a high preponderance to coinfection with other respiratory viral pathogens.^11^ Furthermore, HRV was the predominant virus after SARS‐CoV‐2 was detected either co‐circulating with SARS‐CoV‐2 or circulating alone.^9^ The presence of HRV was assessed, showing that 0.25% of the samples were coinfected SARS‐CoV‐2/HRV. On the other hand, the HRV co‐circulation was 0.8% (Table 1). These results establish HRV co‐circulation and the coinfection with SARS‐CoV‐2. Taken together, these results demonstrate the displacement of seasonal respiratory viruses due to the presence of SARS‐CoV‐2. Despite this displacement, IAV and HRV are still able to keep co‐circulating together with SARS‐CoV‐2 but to a considerably lesser extent in comparison with previous winter seasons.

## 2 DISCUSSION

To gain insights into the potential co‐circulation of the most relevant seasonally circulating respiratory viruses together with SARS‐CoV‐2, a fact on the current COVID‐19 pandemic was that the vast majority of the SARS‐CoV‐2 testing during the April‐July period was indicated only with the presence of symptoms, we arbitrarily selected 200 samples per month (April to July) for a total of 800 NSS from 13 healthcare centers located in the northern zone of Santiago, Chile. In SARS‐CoV‐2 samples, we did not observe any difference between sex; however, there is a 2.27 rate of potential new SARS‐CoV‐2 cases per 1000 habitants between 18 and 40 years old (Table 1).

We detected a high major IR in Location B (Table 1) with a potential of 20 new cases per 1000 habitants, and we observed at least 2‐fold coinfections between SARS‐CoV‐2/IAV or SARS‐CoV‐2/HRV and no coinfections with IBV and RSV, which is in agreement with previously reported data including the southern hemisphere.^5^, ^12^ Furthermore, IAV and HRV were detected from negative SARS‐CoV‐2 samples, whereas no presence of IBV or RSV was obtained even from the negative SARS‐CoV‐2 samples (Table 1). These results demonstrate the displacement of the predominant seasonal respiratory viruses, which have an essential impact during the winter season caused by the high circulation rate of SARS‐CoV‐2. A similar phenomenon was observed after the 2009 influenza A (H1N1) pandemic, which generated a decrease in RSV and IAV H3N2 infections.^13^ The reduction or absence of IAV, IBV, or RSV observed in this study can be explained by the non‐pharmaceutical interventions such as confinement and lockdowns established before the beginning of the winter season in March 2020. A previous report showed that SARS‐CoV‐2 could replace within 3 weeks the seasonal respiratory viruses circulating,^1^ while that HRV coinfections are one of the most commonly observed. However, the impact that HRV infection co‐infecting with other respiratory viruses is still unclear due to inconsistencies among different studies.^11^ The effect of HRV in SARS‐CoV‐2 infection and the clinical outcome is still unknown.

Considering that the vast majority of the SARS‐CoV‐2 testing during the April‐July period was indicated only with the presence of symptoms, potential bacterial infections or coinfections cannot be ruled out in this study. The presence of bacterial infections during the SARS‐CoV‐2 pandemic has been previously reported.^14^, ^15^, ^16^ A previous study identified *S. Pneumoniae, K. pneumoniae, and H. influenza* among the bacteria co‐circulating with SARS‐CoV‐2.^16^ However, the detection of bacteria is beyond the scope of the study.

In conclusion, the data show the impact of SARS‐CoV‐2 over the co‐circulation of seasonal respiratory viruses like IAV, IBV, and RSV in Chile. Our results suggest that the emergence of SARS‐CoV‐2 in addition to different non‐pharmaceutical measures adopted worldwide have a detrimental impact on the circulation at least of seasonal respiratory viruses. Furthermore, our data allow us to foresee the circulation of respiratory viruses in the 2021 winter season in the southern hemisphere.

## CONFLICT OF INTEREST

The authors declare that there are no conflicts of interest associated with this work.

## AUTHOR CONTRIBUTIONS

Conceptualization: Luis A. Alonso‐Palomares, C. Joaquín Cáceres, and Gonzalo P. Barriga

Data Curation: Luis A. Alonso‐Palomares, Rodrigo Tapia, Paulina Aguilera‐Cortés, Aldo Gaggero, Fernando Valiente‐Echeverría, and Gonzalo P. Barriga

Formal Analysis: Luis A. Alonso‐Palomares and Gonzalo P. Barriga

Funding Acquisition: Gonzalo P. Barriga

Investigation: Luis A. Alonso‐Palomares, Rodrigo Tapia, Paulina Aguilera‐Cortés, Santiago Valenzuela, and Gonzalo P. Barriga

Methodology: Luis A. Alonso‐Palomares, C. Joaquín Cáceres, Fernando Valiente‐Echeverría, Aldo Gaggero, RS‐R, and Gonzalo P. Barriga

Project Administration: Luis A. Alonso‐Palomares, C. Joaquín Cáceres, Fernando Valiente‐Echeverría, Aldo Gaggero, Ricardo Soto‐Rifo, and Gonzalo P. Barriga

Resources: Gonzalo P. Barriga

Supervision: Luis A. Alonso‐Palomares, Fernando Valiente‐Echeverría, Aldo Gaggero, Ricardo Soto‐Rifo, and Gonzalo P. Barriga

Validation: Luis A. Alonso‐Palomares, C. Joaquín Cáceres, and Gonzalo P. Barriga

Visualization: Luis A. Alonso‐Palomares, C. Joaquín Cáceres, and Gonzalo P. Barriga

Writing—Original & Draft: C. Joaquín Cáceres, and Gonzalo P. Barriga

Writing—Review & Editing: Luis A. Alonso‐Palomares, C. Joaquín Cáceres, Fernando Valiente‐Echeverría, Aldo Gaggero, Ricardo Soto‐Rifo, and Gonzalo P. Barriga

All authors approved the final version of the manuscript.

Gonzalo Barriga had full data access to all data in this study and takes complete responsibility for the integrity of the data and the accuracy of the data analysis.

## TRANSPARENCY STATEMENT

Gonzalo Barriga affirms that this manuscript is an honest, accurate, and transparent account of the study being reported; that no important aspects of the study have been omitted; and that any discrepancies from the study as planned have been explained.

## Supporting information


**Appendix S1.** Supporting Information.

## Data Availability

The authors confirm that the data support the findings of this study and its supplementary materials.
