# Supplementary material for: Surveillance of seasonal respiratory viruses among Chilean patients during the COVID‐19 pandemic
Source: Health Sci Rep. 2021 Nov 23;4(4):e433. doi: 10.1002/hsr2.433 (PMC8611180; doi:10.1002/hsr2.433)
Supplement: Supplementary file 1 — Appendix S1. Supporting Information. [file HSR2-4-e433-s001.docx]

**Materials and methods**

**Sample selection**

All samples in this study were obtained with nasopharyngeal swabs come from suspected Chilean population infected with SARS-CoV-2 in the north of Santiago de Chile from different health centers (figure 1). The samples were collected in 2 mL of RNA-shield media (GenoSUR) and store at room temperature until its analysis for SARS-CoV-2 detection.

**RNA extraction and Identification of respiratory viruses by RT-qPCR**

The RNA extraction was made using the Total RNA Purification Kit (Norgen Biotek CORP); following the manufacturing procedure, the RNA was a store at -80°C, which was used to perform RT-qPCR.

All samples were analyzed using a specific primer (Supplementary table 1) for SARS-CoV-2, IAV, IBV, RSV, and HRV. All sequences have been validated, and their use is a typical procedure to detect respiratory viruses from the World Health Organization (WHO). The viral genome detection was made using LightCycler® Multiplex RNA Virus Master (Roche) following the manufacturing procedure. The amplification and analysis plot was made in a QuantStudio3 Real Time PCR System 96 wells (Thermo Fisher Scientific). The HRV and IBV detection was performed by RT-PCR final point using specific primers, the retrotranscription step was made using SuperScript IV Reverse Transcriptase (Thermo Fisher Scientifics) and PCR was using GoTaq® DNA polymerase (Promega), the genome of HRV was visualized in agarose – Seakem LE Agarose (LONZA) at 2% applying a voltage of 80 Volts for 30 minutes.

**Supplementary Table 1.- Primers Sequence for SARS-CoV-2, IAV, IBV, RSV and HRV**

| **Virus** | **Forward** | **Reverse** |
| --- | --- | --- |
| SARS-CoV-2 | 5’ ATGAGCTTAGTCCTGTTG 3’ | 5’ CTCCCTTTGTTGTGTTGT 3’ |
| IAV, | 5’ GACCRATCCTGTCACCTCTGA C 3’ | 5’ AGGGCATTYTGGACAAAKCGTCTA 3’ |
| IBV, | 5’ GGAGCAACCAATGCCAC 3’ | 5’ GTKTAGGCGGTCTTGACCAG-3’ |
| RSV | 5’ AACAGATGTAAGCAGCTCCGTTATC 3’ | 5’-CGATTTTTATTGGATGCTGTACATTT 3’ |
| aHRV | 5´CAAGCACTTCTGTTTCCC 3´ | 5´CACGGACACCCAAAGTAGT 3´ |
